# Non-invasive skin autofluorescence, blood and urine assays of the advanced glycation end product (AGE) pentosidine as an indirect indicator of AGE content in human bone

**DOI:** 10.1186/s12891-019-3011-4

**Published:** 2019-12-27

**Authors:** Yoshikuni Kida, Mitsuru Saito, Akira Shinohara, Shigeru Soshi, Keishi Marumo

**Affiliations:** Department of Orthopaedic Surgery, Jikei University School of Medicine, 3–25–8, Nishi-Shinbashi, Minato-ku, Tokyo, 105–8461 Japan

**Keywords:** Collagen, Advanced glycation end products (AGEs), Pentosidine, Bone quality, Biomarker

## Abstract

**Background:**

Bone mineral density (BMD) measurements are widely used to assess fracture risk. However, the finding that some fracture patients had high BMD together with the low contribution of drugs to osteoporosis suggests that bone strength factors other than BMD contribute to bone quality. We evaluated the amount of advanced glycation end products (AGEs) by non-invasive assays of serum and urine as well as by skin autofluorescence to measure the levels of a representative AGE, pentosidine, to investigate whether pentosidine can serve as an indirect indicator of AGEs formation in bone collagen.

**Methods:**

A total of 100 spinal surgery patients without fragility fracture (54 males and 46 females) treated at our hospital were enrolled. The amount of pentosidine in blood, urine, skin and bone (lumbar lamina) samples from these patients was measured. AGE accumulation was assessed by measuring skin autofluorescence. We examined the correlation between pentosidine content in tissues and body fluid, as well as skin AGEs with age, height, body weight, BMI, and estimated glomerular filtration rate (eGFR).

**Results:**

A significant age-related increase in pentosidine levels in tissues was observed, while there was a significant negative correlation between tissue pentosidine and eGFR. The amount of skin pentosidine was significantly and positively correlated with pentosidine content of the bone in those under 50 years of age. Urine pentosidine also correlated positively with bone pentosidine and skin pentosidine, but only in females. The total amount of AGEs in skin did not correlate with bone pentosidine.

**Conclusion:**

In this study, the strong correlation between the pentosidine content in each sample and eGFR may indicate that renal dysfunction with advancing age increases oxidative stress and induces AGEs formation in collagen-containing tissues. The correlation of skin pentosidine concentration and eGFR, with AGEs formation in bone collagen suggests that pentosidine would be a useful indirect index of decreased bone quality. Skin AGEs estimated by autofluorescence in clinical situations may not be suitable as an indirect assessment of bone quality. Because urine pentosidine correlated positively with bone pentosidine and skin pentosidine in females, urine pentosidine may be a candidate for an indirect assessment of bone quality.

## Background

At present, the measurement of bone mineral density (BMD) is widely used for the evaluation of bone fracture risk in daily clinical practice. However, clinical data from bone fracture cases suggest, that despite high BMDs and the potential of drugs used to increase BMD by approximately 4–30% in patients requiring treatment for osteoporosis to reduce bone fracture risk, the importance of *bone quality—*a determinant of bone strength besides bone density—has been identified [1, 2]. The determinants of bone quality include structural and material properties of the bone. Structural properties are regulated by bone turnover, whereas the determinants of bone material properties such as advanced glycation end products (AGEs) formation in bone collagen are not only dependent on tissue turnover but also on the extent of oxidative stress and glycation [1, 3, 4].

We have previously reported that the crosslinks formed to connect collagen molecules, which account for 50% per bone unit cubic volume, play an important role in determining bone properties, particularly bone strength, given that structure is integral to bone quality [1]. Collagen crosslinks are classified on the basis of enzyme-dependent crosslinks which facilitate the dynamic function of bone, calcification (enzymatic crosslink formation), and AGEs which can cause brittle bone (nonenzymatic crosslink formation) [3]. Pentosidine is a well-established intermolecular cross-linking AGE and is used as a surrogate marker for total AGEs [1, 3]. The content of pentosidine in tissues such as bone and skin has a significant positive association to the total AGEs in each tissue [1, 3].

We elucidated that in primary osteoporosis patients with hip fractures, an excessive accumulation of pentosidine and limited enzymatic crosslink formation in early stages of bone formation can contribute to brittle bones [5]. We previously reported excessive formation of pentosidine in the bone from ovariectomized rabbits and monkeys as well as from patients with osteoporotic hip fractures using a single column high performance liquid chromatography, which we developed independently [1, 5–9]. Pentosidine has also been implicated as an independent risk factor for hip and vertebral fractures in other studies [10, 11]. Furthermore, excessive formation of pentosidine is an independent determinant of decreased bone strength demonstrated by multiple regression analysis using human, monkey, and rabbit specimens [1, 5–8]. Therefore, the establishment of a noninvasive methodology to evaluate bone material property is needed in the clinical situation. Currently, it is unknown whether blood and urinary concentrations of pentosidine are surrogate measures of the pentosidine content of bone collagen [12–14]. Thus, in this study, bone, skin, urine, and blood were collected from patients and the correlativity among the samples in terms of pentosidine concentrations was investigated. Because skin collagen ages in a similar manner to bone collagen, it attracted our attention as a useful tissue to explore further. Skin AGEs were evaluated using a noninvasive skin autofluoroscope or AGE Reader (Diagnoptics Technologies BV, Netherlands) and its correlation with the level of pentosidine, a representative structure of AGEs, was evaluated to investigate if it is useful as an indirect evaluation method of AGEs formation of bone collagen. The aim of this study is to assess noninvasive methods to measure AGEs in skin, serum and urine to determine whether they may be useful tools for indirect estimation of pentosidine in the bone.

## Methods

Informed consent was obtained from patients with spine disease before surgery, and an ethical committee approved the study. Among the spinal surgery cases which were treated at our hospital, blood, urine, skin, and bone (lumbar lamina) samples were collected from 100 cases (54 males and 46 females). There were no prevalent osteoporotic fractures in these patients. Patients undergoing hemodialysis, or those who had diabetes mellitus or rheumatoid arthritis were excluded. Patients with diabetes mellitus or rheumatoid arthritis were excluded because these patients received medical treatments such insulin, Methotrexate, steroids, and biological agents. It is reported that these medical treatments themselves affect both the levels of oxidative stress and AGEs formation [15, 16]. Serum creatinine (Cr) and serum calcium were measured by automated techniques at the central laboratory of our hospital. Glomerular filtration rate (GFR) was estimated using the Cockcroft-Gault equation. Measurements of pentosidine in bone and skin samples were performed using our previously reported method that involves conversion of pentosidine concentrations per collagen molecule [9]. Briefly, all tissues were washed with phosphate-buffered saline and powdered in a liquid nitrogen-cooled freezer mill. The specimens were hydrolyzed in 6 N HCl. Hydrolysates were then analyzed for crosslink and hydroxyproline content on a Shimadzu LC9 HPLC fitted with a cation exchange column (0.9Ed Aa pack-Na, Jasco, Tokyo, Japan) linked to an online fluorescence flow monitor (RF10AXL, Shimadzu, Kyoto, Japan). The amount of collagen was assumed to be 7.5 times the measured hydroxyproline weight, based on a molecular weight of 300,000. The resulting data were used to calculate crosslink values expressed as mol/mol of collagen. Pentosidine amounts were detected by natural fluorescence. Serum pentosidine was measured using the ELISA method (FUSHIMI Pharmaceutical Co Ltd., Kagawa, Japan.) [17]. In brief, pentosidine molecules bound to plasma proteins were released by treatment of samples with pronase at 55 °C for 1.5 h. The mixture was then heated in boiling water for 15 min to inactivate the enzyme. Pentosidine antibody and pretreated samples were added to each well and incubated at 37 °C for 1 h after washing. Peroxidase-labeled goat anti-rabbit IgG polyclonal antibodies were added and incubated for 1 h at room temperature before a color development reagent was added to each well. The reaction was stopped 10 min later. The absorbance was measured within 10 min at 450 and 630 nm (main and reference wavelength, respectively). The inter- and intraassay coefficients of variations of absorbance were 6.6 and 8.0%, respectively. Urine pentosidine levels were measured by HPLC using a method described by Yoshihara et al. after extraction of urine samples using a CF-11 cellulose column to yield hydrolysates of urine samples [18]. Urine pentosidine levels were corrected with respect to urinary creatinine. The measurable range for this assay system was 1.7–55.6 pmol/ml pentosidine, and the recovery of the standard ranged between 84.9 and 108%. The inter- and intraassay variances for pentosidine were 5.5%. Variations in pentosidine levels between samples from 24 h urine and the first and second voids were minimal. AGE accumulation in the skin was assessed by measuring skin autofluorescence (AF) using an AGE Reader [19–21]. Skin AF was measured at the volar side of the arm. All measurements were performed at room temperature in a dark environment, within 30 s, without pain or impairment of the skin. Autofluorescence was calculated by an automated observer-independent analysis by dividing the average light intensity of the emission spectrum (300–600 nm) by the average light intensity of the excitation spectrum (300–420 nm). Reproducibility of this measurement was shown by a mean coefficient of variation of around 5%.

The difference in characteristics between genders was evaluated using ANOVA, and the correlation for each parameter was tested using Spearman’s rank correlation coefficient. All tests were two-sided, and *p* <  0.05 was considered significant.

## Results

**Fig. 1:**
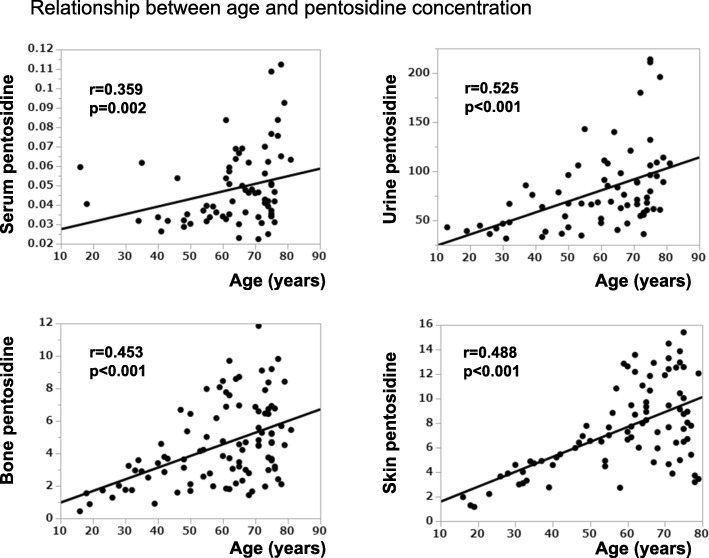
Changes in pentosidine with ageing

Baseline characteristics of patients, including age, height, body weight, and BMI are summarized in Table 1, in addition to measurements of eGFR, AGEs in skin, and pentosidine contents and concentrations in tissues and body fluids, respectively. There were no differences in these parameters between male and female cases. Table 2 shows the correlations between age, BMI, eGFR, and tissue pentosidines. Significant positive correlations were observed between age and pentosidine contents, and also between age and AGEs in skin (Fig. 1 and Table 2). Pentosidine at each site was also negatively correlated with eGFR. When the patient groups were divided into age groups below 50 years and over 50 years, bone pentosidine, skin pentosidine and skin AGE in the under 50 years group showed significant correlation positively with age (*r* = 0.716, *p* <  0.001; *r* = 0.872, *p* <  0.001; *r* = 0.627 *p* = 0.029, respectively) and negatively with eGFR (*r* = − 0.592, *p* = 0.002; *r* = − 0.760, *p* <  0.001; *r* = − 0.832 *p* = 0.001, respectively). On the contrary, serum pentosidine correlated positively with age and negatively with eGFR in those over 50 years (*r* = 0.320, *p* < 0.015; *r* = − 0.393, *p* < 0.002 respectively).

**Table 1:** Characteristics of surgical cases from which bone samples were collected

Characteristics	Cases *N* = 100	Male	Female	*p*
Age (years)	58.5 ± 17.2	58.2 ± 17.4	58.9 ± 17.1	0.852
Gender, n (%)				
Male	54 (54%)			
Female	46 (46%)			
Height (cm)	159.7 ± 9.2	164.9 ± 7.7	153.6 ± 6.9	< 0.001
Body weight (kg)	60.7 ± 11.4	65.7 ± 10.0	54.9 ± 10.1	< 0.001
BMI (kg/m^2^)	23.7 ± 3.4	24.1 ± 2.9	23.2 ± 3.9	0.210
eGFR (mL/min/1.73 m^2^)	83.4 ± 26.1	83.5 ± 23.4	83.2 ± 29.3	0.952
Serum pentosidine concentration (μg/mL)	0.05 ± 0.02	0.05 ± 0.02	0.05 ± 0.02	0.176
Urinary pentosidine concentration (pmol/mg Cr)	79.8 ± 40.8	71.7 ± 39.3	90.8 ± 40.8	0.056
Bone pentosidine (mmol/mol of collagen)	4.5 ± 2.5	4.8 ± 2.5	4.1 ± 2.4	0.202
Skin pentosidine (mmol/mol of collagen)	7.5 ± 3.5	6.9 ± 3.1	8.4 ± 3.8	0.051
Skin AGE reader (AU)	2.4 ± 0.5	2.4 ± 0.5	2.5 ± 0.5	0.488

Data are the mean ± standard deviation, unless otherwise indicated

*AGE* Advanced glycation end products, *BMI* Body mass index, *Cr* Creatinine, *eGFR* Estimated glomerular filtration rate

*p*-value: male versus females

**Table 2:** Correlation of pentosidine content with patient characteristics

		Pentosidine concentration								Skin AGE content	
		Serum		Urine		Bone		Skin			
		*r*	*p*	*r*	*p*	*r*	*p*	*r*	*p*	*r*	*p*
Patient Parameter	Age (total)	0.359	0.002	0.525	< 0.001	0.452	< 0.001	0.488	< 0.001	0.462	< 0.001
	male	0.202	0.245	0.430	0.020	0.475	0.001	0.415	0.011	0.482	0.006
	female	0.490	0.003	0.635	< 0.001	0.485	< 0.001	0.527	< 0.001	0.456	0.013
	< 50 years	−0.361	0.276	0.238	0.358	0.716	< 0.001	0.872	< 0.001	0.627	0.029
	> = 50 years	0.320	0.015	0.261	0.065	0.135	0.259	0.029	0.825	0.231	0.115
	BMI(total)	0.019	0.877	0.070	0.572	0.155	0.135	−0.067	0.551	−0.088	0.510
	male	0.037	0.837	0.260	0.173	0.059	0.705	−0.073	0.671	0.095	0.616
	female	−0.081	0.649	−0.107	0.518	0.234	0.095	0.096	0.527	−0.292	0.125
	< 50 years	−0.322	0.364	−0.196	0.451	0.196	0.371	−0.179	0.450	−0.164	0.631
	> = 50 years	0.037	0.782	0.023	0.874	0.110	0.357	−0.140	0.280	−0.102	0.489
	eGFR (total)	−0.394	0.001	−0.370	0.002	−0.358	< 0.001	−0.410	< 0.001	− 0.353	0.006
	male	−0.328	0.054	−0.314	0.098	−0.402	0.007	−0.356	0.031	−0.366	0.043
	female	−0.473	0.005	−0.441	0.005	−0.376	0.006	−0.409	0.004	−0.332	0.079
	< 50 years	0.237	0.483	−0.031	0.907	−0.592	0.002	−0.760	< 0.001	−0.832	0.001
	> = 50 years	−0.393	0.002	−0.097	0.499	−0.078	0.518	−0.069	0.593	−0.131	0.375

**Fig. 2:**
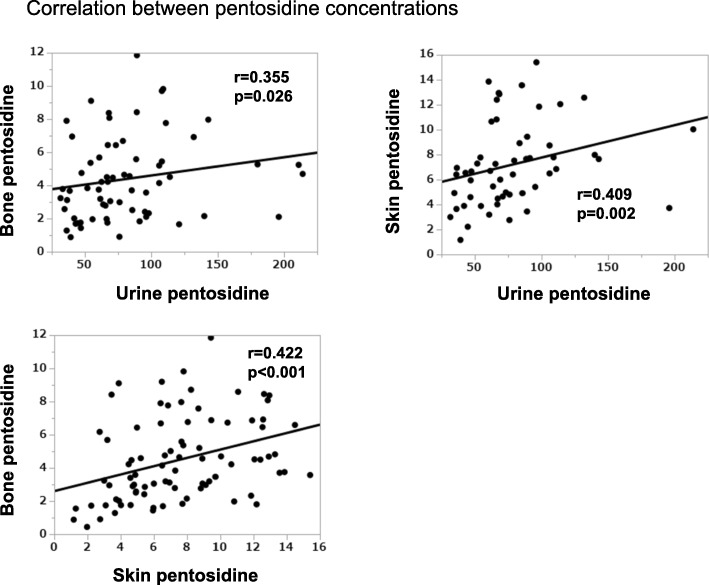
Correlation between bone, skin and urine pentosidine

Correlation between pentosidine in different tissues as well as skin AF was calculated and shown in Fig. 2 and Table 3. Total and female urinary pentosidine concentrations were significantly correlated with the concentration in bone (*r* = 0.355, *p* = 0.026; *r* = 0.346, *p* = 0.033, respectively) and skin (*r* = 0.409, *p* = 0.002; *r* = 0.482, *p* = 0.005, respectively). Moreover, pentosidine concentration in the bone was significantly correlated with skin content (*r* = 0.422, *p* < 0.001). Although AGEs in the skin were significantly correlated with skin pentosidine content (*r* = 0.314, *p* = 0.008), the correlativity between AGEs in skin and the pentosidine content of bone was not significant (*r* = 0.216, *p* = 0.104). Again, when the patients were divided into different age groups, skin pentosidine showed significant correlation with bone pentosidine under 50 years (*r* = 0.696, *p* < 0.001).

**Table 3:** Correlation between pentosidine concentrations

		Pentosidine concentration						Skin AGE content	
		Urine		Bone		Skin			
		*r*	*p*	*r*	*p*	*r*	*p*	*r*	*p*
Pentosidine concentration	Serum (total)	0.290	0.066	−0.031	0.799	0.027	0.842	0.180	0.180
	male	0.181	0.447	−0.075	0.667	−0.001	0.995	0.289	0.121
	female	0.378	0.091	−0.023	0.898	0.211	0.271	0.136	0.500
	< 50 years	1.000	< 0.001	−0.638	0.035	−0.400	0.286	−0.300	0.433
	> = 50 years	0.162	0.333	−0.027	0.840	−0.009	0.954	0.131	0.375
	Urine (total)			0.355	0.026	0.409	0.002	0.304	0.060
	male			0.379	0.051	0.229	0.306	0.038	0.878
	female			0.346	0.033	0.482	0.005	0.435	0.056
	< 50 years			−0.064	0.820	0.335	0.263	−0.200	0.747
	> = 50 years			0.122	0.399	0.252	0.112	0.205	0.245
	Bone (total)					0.422	< 0.001	0.216	0.104
	male					0.406	0.013	0.215	0.255
	female					0.544	< 0.001	0.320	0.097
	< 50 years					0.696	< 0.001	0.427	0.190
	> = 50 years					0.095	0.462	0.121	0.416
	Skin (total)							0.314	0.008
	male							0.169	0.419
	female							0.416	0.043
	< 50 years							0.733	0.025
	> = 50 years							0.119	0.466

## Discussion

In this study, we evaluated whether the measurement of AGEs, pentosidine in serum by ELISA or urine by HPLC or skin AGEs by autofluorescence, is an indirect indicator of the levels of pentosidine in bone collagen.

Sell et al. and Odetti et al. have reported evidence of an exponential increase in the accumulation of pentosidine in skin collagen with advancing age [22, 23]. We also reported that pentosidine concentration in the human bone gradually increases with age [9]. Furthermore, we also showed that pentosidine concentration in bone correlated significantly and positively to total AGEs including pentosidine [1]. Pentosidine in individuals without fractures was measured in this study, as previous cadaveric reports by Nyman et al. and Hernandez, Bank et al. have shown that pentosidine increase contributes to bone weakness in the form of decreased energy dissipation and ductility, even in those without fractures [24, 25]. Wang et al. have also shown age-dependent pentosidine difference in the middle aged and elderly groups [26]. Pentosidine levels vary more in the elderly, and pentosidine correlations were evaluated in two age groups: those under the age of 50, and those who are 50 or over. Statistically, significance was lost in certain categories as there were fewer cases of those under the age of 50. In addition to this, pentosidine values showed greater variance in those who were 50 or above. This may be due to the multifactorial contribution for AGEs accumulation. With many of the factors contributing to AGEs accumulation associated with age, it is perhaps unsurprising that AGEs accumulation does not increase in a uniform linear pattern, and with greater individual variance due to different lifestyles and risk factors [27].

Our findings also showed that urinary pentosidine concentrations were significantly and positively correlated with the accumulation of pentosidine in bone and skin. Based on these findings, because the pentosidine content among samples were correlated, it suggests that AGEs formation in body tissues may to an extent commensurate with the degree of oxidative stress and hyperglycemia-induced glycation that one is exposed to [27]. In this study, the significant correlation between the pentosidine content of each sample and eGFR may indicate that renal dysfunction with advancing age increases oxidative stress and induces the formation of AGEs in collagen-containing tissues (Additional file 1). Pentosidine is reflective of the long-term accumulation of AGEs, whereas eGFR is variable and may only reflect the renal function at the time of investigation. However, eGFR was investigated for all patients prior to surgery, and is likely to represent their usual renal function. Therefore we assumed that a low eGFR represents a chronic renal impairment and also a long-term increase of oxidative stress, thus contributing to accumulation of AGEs, as opposed to a temporary renal impairment [4, 28]. It is conceivable that the strongest correlation of skin pentosidine and eGFR with the formation of AGEs in bone collagen together represent a useful indirect index for decreased bone quality and thus demonstrating its material property.

In terms of assessment of the material property of bone, the estimation of urine pentosidine concentration may be useful in a clinical setting. Wang et al. showed that age-related accumulation of pentosidine is an independent determinant of bone strength using bone specimens without osteoporotic fractures [26]. We also demonstrated that pentosidine concentration in human vertebral and femoral bones increased with age despite the lack of osteoporotic fractures [3, 9]. However, pentosidine concentration of bones from patients with osteoporotic hip fractures was significantly higher than in the age-matched subjects without fractures [5]. Furthermore, a high urinary concentration of pentosidine is an independent risk factor for bone fracture, even when the value is corrected for eGFR, therefore, urine pentosidine should not be considered the same as eGFR in terms of fracture risk [12, 14, 29]. In a longitudinal study investigating osteoporotic incidence of vertebral fractures in a 5-year prospective study in 432 elderly Japanese women, we showed that high urine pentosidine is a risk factor independent of age and eGFR [13]. The subjects in this study have no osteoporotic fractures, thus, a normal age-related gradual increase of pentosidine in the bone does not necessary increase fracture risk. However, an excessive accumulation of pentosidine that is greater than the expected physiological accumulation with age and renal function may lead to an increased fracture risk. Therefore, further studies are warranted to confirm the relationship between skin, urine and bone pentosidine concentrations and age in patients with osteoporotic fractures.

This study has revealed that skin pentosidine is most reflective of bone pentosidine, especially in those under 50 years of age (Table 3). Thus skin pentosidine may provide the most accurate representation of bone pentosidine. The studies also assessed the validity of skin AF as a non-invasive tool for assessing bone pentosidine. Although skin AF showed a positive correlation with skin pentosidine as expected, no significant correlation was not obtained with bone pentosidine.

This study has some limitations. Firstly, it is known that the concentration of pentosidine in serum and urine is influenced by renal function and increases with advancing age as mentioned above. Furthermore, for patients exposed to excessive systemic oxidative stress, there is a strong possibility that AGEs will be simultaneously formed in various collagenous tissues throughout the body, which can lead to arteriosclerosis when blood vessels are implicated and bone fractures when bone quality deteriorates by glycation. Secondly, measurement of pentosidine in various tissues may not reflect the total amount of AGEs. However, pentosidine is a well-established intermolecular crosslinking AGE [3, 5, 7, 30–32] and serves as a surrogate marker of the total level of AGEs [1, 31, 32]. Therefore, it can be assumed that there is at least a direct correlation between skin pentosidine and the total AGEs content of the tissue.

## Conclusion

Skin pentosidine is effective as a marker of AGEs accumulation in the bone in those under the age of 50. Urine pentosidine levels also correlated significantly and positively with bone pentosidine especially in females. Thus, the examination of skin and urine pentosidine in clinical settings may be suitable indirect indicators of poor bone quality.

## Additional file

**Additional file 1:** Correlation between eGFR and age.

## Data Availability

The datasets analyzed during the current study are available from the corresponding author on reasonable request.

## Abbreviations

AF: Autofluorescence
AGEs: Advanced glycation end products
BMD: Bone mineral density
BMI: Body mass index
eGFR: Estimate glomerular filtration rate
ELISA: Enzyme-linked immunosorbent assay
HPLC: High performance liquid chromatography
