# Supplementary figures and images for: Non-invasive skin autofluorescence, blood and urine assays of the advanced glycation end product (AGE) pentosidine as an indirect indicator of AGE content in human bone
Source: BMC Musculoskelet Disord. 2019 Dec 27;20:627. doi: 10.1186/s12891-019-3011-4 (PMC6933723; doi:10.1186/s12891-019-3011-4)

## Slide 1
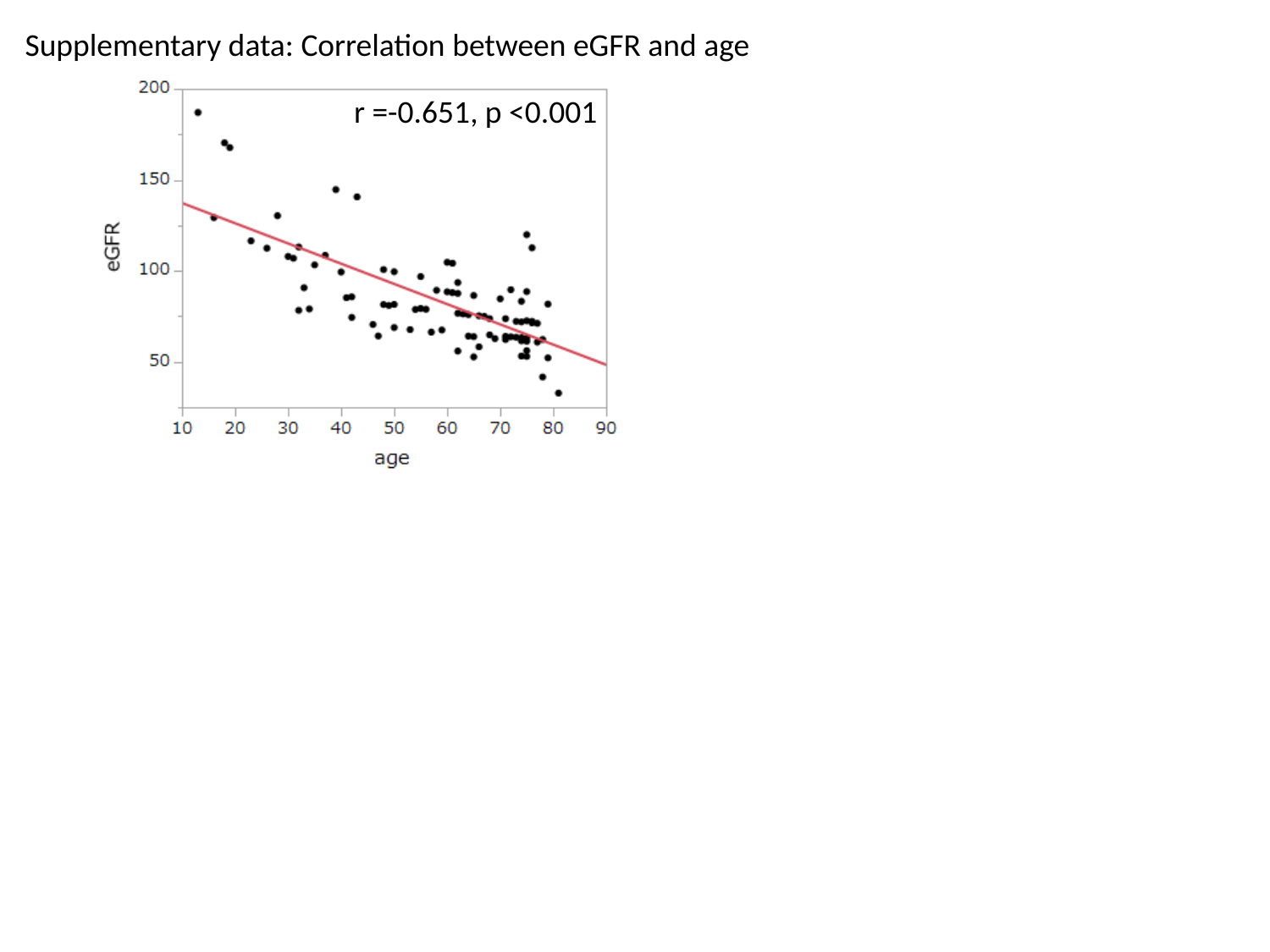

Supplementary data: Correlation between eGFR and age
r =-0.651, p <0.001

Supplement: Supplementary file 1 — Additional file 1: Correlation between eGFR and age. [file 12891_2019_3011_MOESM1_ESM.pptx]
